# Soy, Soy Foods and Their Role in Vegetarian Diets

**DOI:** 10.3390/nu10010043

**Published:** 2018-01-05

**Authors:** Gianluca Rizzo, Luciana Baroni

**Affiliations:** 1 Via Venezuela 66, 98121 Messina, Italy; 2 Primary Care Unit, Northern District, Local Health Unit 2, 31100 Treviso, Italy; luciana_baroni@yahoo.it

**Keywords:** soy foods, vegetarian diets, phytoestrogens, isoflavones, protein quality, meat analogues, thyroid health, endocrine disruptor, selective oestrogen receptor modulator, cancer

## Abstract

Soy is a basic food ingredient of traditional Asian cuisine used for thousands of years. In Western countries, soybeans have been introduced about a hundred years ago and recently they are mainly used for surrogate foods production. Soy and soy foods are common nutritional solutions for vegetarians, due to their high protein content and versatility in the production of meat analogues and milk substitutes. However, there are some doubts about the potential effects on health, such as the effectiveness on cardiovascular risk reduction or, conversely, on the possible disruption of thyroid function and sexual hormones. The soy components that have stimulated the most research interest are isoflavones, which are polyphenols with estrogenic properties highly contained in soybeans. In this review, we discuss the characteristics of soy and soy foods, focusing on their nutrient content, including phytoestrogens and other bioactive substances that are noteworthy for vegetarians, the largest soy consumers in the Western countries. The safety of use will also be discussed, given the growing trend in adoption of vegetarian styles and the new soy-based foods availability.

## 1. Introduction

In the last decades, vegetarian diets have become more widespread among the population. The reasons for this choice are different and include mainly ethical, ecological and health aims [1,2,3].

Shift from omnivore to vegetarian diet implies the intake of nutrients from plant sources with sustainable and well-planned dietary schemes [4]. Some supplements are needed to ensure adequate nutrition if the diet falls to guarantee correct essential nutrients. Overall, the most important and accepted supplement is the vitamin B12 supplementation, scarcely represented in plant foods [4]. Also protein quality has often been a cause for debate because of different amino acid pattern of plant foods in relation to animal ones, which has been speculated to affect protein synthesis; this concept led to the definition of “high quality protein” referred to proteins from animal foods [5]. However, increasing variety and quantity of plant foods may overcome this issue and may guarantee the intake of all essential amino acids needed for an adequate nutrition plan [6,7]. Essential amino acid consumption needs more emphasis in children and individuals engaging in sport activity, with specific increased turnover in body proteins [8,9,10].

Increasing interest over the last years has been paid to protein from plant sources: there is evidence that individuals consuming foods high in vegetable proteins (i.e., legumes) have lower risk of cardiovascular disease and other metabolic disorders [11,12]. Among others, protein quality of soy beans is one of the most attractive reasons for the interest in soy and soy foods among vegetarians [13]. With the growing adoption of vegetarian lifestyles, a great variety of soy-based food products have become more available in grocery stores; besides the market request, a reason for such popularity may depend on the nutritional and versatile properties of soy beans, which are suitable for food technological transformations [14]. Soy beans are used for the production of several analogues and surrogates of meat and dairy products that might be used as alternatives especially during transition to vegetarian diet [13].

High protein content, together with lower carbohydrate content, characterizes soy as an unique vegetable protein source compared to other legumes [10]. Nutrient composition of some legumes, including soy beans, is summarized in Table 1.

Furthermore, the production of protein sources from plants can be a sustainable option for reducing the ecological exploitation of natural sources during cattle breeding for meat production [13,16].

Besides macro- and micronutrients, soy contains high concentrations of phytoestrogens, polyphenols with similar molecular structure as endogenous oestrogens, which still raise doubts about safe use, especially at high dose [17]. In Asian regions, soy is often used for several traditional cuisine dishes. Soy beans can be blended and heated to extract soy milk that can also be treated with magnesium chloride or calcium sulphate curd to get tofu. In addition, different fermentation treatments are useful in order to get natto, tempeh, soy sauce and sufu [18].

The high consumption of soy foods and, consequently, isoflavones in Asian populations dissipated critical concerns on safety; however, their use in Western countries and their role on human health is still debatable [19,20,21].

In this review we discuss worldwide consumption, nutrient composition and bioactive compounds in soy and soy foods. Furthermore, we discuss current evidence of the possible effects on human health and safety. These arguments are relevant for Western countries with growing soy consumption, especially by vegetarians [22].

A non-systematic literature search of MEDLINE (http://www.ncbi.nlm.nih.gov/pubmed) and EMBASE (http://www.embase.com) was conducted by using the following subject headings/key words or MeSH terms where available: (a) ‘soy’ OR ‘soy foods’ OR ‘soya’ OR ‘soy beans’ OR ‘glycine max’ OR ‘soy products’ OR ‘meat analogues’ OR ‘meat substitutes’ OR ‘meat alternatives’ OR ‘texturized vegetable protein’ OR ‘soy protein’ OR ‘soy milk’ OR ‘traditional soy foods’; and (b) ‘vegetarian’; and (c) ‘consumption’ OR ‘protein quality’ OR ‘isoflavones’ OR ‘phytochemicals’ OR ‘nutrients’ OR ‘bioactive compounds’ OR ‘cancer’ OR ‘thyroid’ OR ‘sex hormones’ OR ‘health benefits’ OR ‘chronic diseases.’ The search was extended to references within.

## 2. Soy Consumption

Major worldwide soy consumers are historically identified in Asian populations. The great consumption in the Asian regions depends on several traditional Asian foods that use soy beans as the main ingredient [23,24,25,26,27]. However, in the last decade, consumption of soy foods in Western countries has grown with the increase in vegetarian lifestyle and healthy perception of soy intake [28].

Total grams, soy protein or soy isoflavone intake can be used as indexes of soy consumption, due to relevance of protein and isoflavone fractions and relative variability in soy foods. The intake of 100.6 g per day, 8.7 g per day and 39.6 mg per day of soy, soy protein and isoflavones, respectively, have been reported among Chinese women from Shanghai [23]. In perimenopausal cohort of women from Hong Kong, median intake of soy protein was 4.5 g (despite with great variability across individuals) [29]; however, other studies reported up to 17 g per day of soy protein intake in Shanghai women with more plant based dietary habits [30]. Among the adult population from Japan, mean soy protein intake ranged between 6 to 11 g per day with isoflavones intake of 23–54 mg per day [31]. Mean soy, soy protein and soy isoflavone intake in 47 Japanese prefectures between 1980 and 1985 has been reported to be 66.8 g, 6.5 g and 27.8 mg, respectively [32]. In Japan, median intake of daidzein and genistein (about 90% of isoflavone fractions) [33] have been reported to be between 9 and 12 mg per day and 15 and 20 mg per day, respectively [24]. However, decreased intake of soy protein in respect to animal protein occurred in the last decades because adoption of more Westernized habits in Asian countries [31]. In Japanese men, the plasma isoflavone concentration of genistein and daidzein was 493 and 283 nM, respectively, whereas in British men (reporting no soy consumption) concentration of these components was 33 and 18 nM, respectively [34,35,36]. In Korea isoflavone intake has been estimated to be about 15 mg per day with about 21 g of traditional soy foods daily per capita [25]. Women from Samsung Medical Centre at Sungkyunkwan University in Seoul, Korea, recruited for a study of correlation between breast cancer risk and tofu intake from case-control studies, reported median daily intake of 7.4 and 8.5 g of soy protein among cases and controls, respectively [37]. Median daily tofu intake was between 27 and 36 g.

In USA and Europe, the intake of isoflavones was less than 3 mg per day [38,39]. Usually, isoflavone intakes were estimated to be less than 1 mg per day per capita in Western countries but with a range between 0.3–4.5 mg per day in European countries and about 1 to 3 mg per day in USA [22,36,40,41,42]. Vegetarians had the major intake among the Western people, with 7–12 mg per day of total isoflavones [22]. In 2002, soy intake was calculated in the 35,955 adults of the European Prospective Investigation into Cancer and Nutrition (EPIC) study on 10 European countries (the UK, The Netherlands, Sweden, Spain, Norway, Italy, Greece, Germany, France and Denmark) [43]. Soy consumption was very low among the European countries, with a total of 681 (1.9%) participants reporting to consume soy or soy foods and with the higher contribution from UK. Interestingly, a subgroup of individuals of the UK-EPIC arm, classified as following a health-conscious lifestyle (HHL) (defined as non-meat eaters, fish eaters, vegetarians, or vegans) showed the highest soy intake compared to the whole sample: specifically, compared to 1% of males and 2% of female among the UK participants in the general population, 35% of males and 24% of females among HHL reported soy consumption. UK HHL consumed up to 149 g per day of soy dairy substitutes with an isoflavone intake of 15–30 mg per day [23,24,25]. In another British study of women from the Oxford-EPIC cohort, including vegetarian and vegan subgroups, it has been reported an intake up to 30 g per day of soy protein and 130 mg per day of isoflavones, with most of the highest soy intake in vegetarians or vegans [44]. More recently, a pan-European investigation on polyphenol consumption showed an overall general north-south gradient, with higher intake in Northern European countries. Intake of isoflavones has been reported to be very low in populations living in the Mediterranean area [45]: on average, population living in countries such as Greece, Spain and Italy showed a range of intake from 10 μg/day to up to approximately 1 mg/day [46,47]; interestingly, compared to other European countries, the main sources of phytoestrogens in Mediterranean countries have been reported to be legumes (i.e., beans and peas), nuts, fruits, vegetables, grains and seeds [48,49].

In the US, the community of devotees to the Seventh-day Adventist Church is a precious source of epidemiological data for the common adherence to vegetarian, ovo-lacto vegetarian and vegan diets. Furthermore, it is a cohort with homogeneous habits, very useful for the definition and comparison of vegetarian nutritional intakes [50,51]. Among soy consumers in Adventist Health Study-2 (from USA and Canada), mean daily soy protein intake was 9.25 g and 8.42 g, assessed with 24 h recall and FFQ, respectively [52]. In postmenopausal women, isoflavone intake ranged between 0 and about 1 g per week [53].

The wide variability of soy foods and soy intakes among diverse populations provides a challenge to define mean consumption; nevertheless, an intake of 10–12 g per day of soy protein seems to be convincing among vegan, with about half of this amount among non-vegan vegetarian [54]. A summary of soy intake estimates is presented in Table 2.

## 3. Soy Nutrients and Bioactive Compounds

Soy, *Glicine max* (L.), is a plant of Asian origin belonging to Fabaceae family. Worldwide producers of soy bean are USA, Brazil, Argentina, China and India with world production volumes of 35%, 28%, 17%, 4% and 3%, respectively [55]. In the soy industry, after oil extraction, a consistent fraction is used for the production of fodder for livestock [56]. Italy is the European country with the highest production of soy beans, with 933,140 tons per year [55], based on GMO free crops [57] The Minister of Agriculture and Forestry in consultation with the Minister for the Environment, Land and Sea and the Minister of Health to European Commission after actuation of European directive 2015/412 permits member state to adopt measures restricting or prohibiting the cultivation of GMOs in all or part of its territory [58]. GMOs are genetically modified organisms that are specially used for crops to maximize commodities production for food and feed. In Europe fifteen type of GM soy crops are regular registered and allowed [59]. There are some doubts raised from animal studies and in particular upon concomitant use of GMO and herbicides [60]. Even if differences between GM and non-GM soybeans were proposed in relation to health efficacy of bioactive compounds [61], actually there are not sufficient data to definitively consider the impact of GMOs on human health but results from scientific literature prompt to a safe profile of GMOs [62].

Different climatic conditions and different cultivation practices lead to variable bean dimension and isoflavone content [63]. The isoflavone content in soybean from different locations is summarized in Table 3.

The health effects of soy protein stimulated the interest of scientific research [65,66]. Compared to other dietary sources, soy has by the highest content of isoflavones, which has been shown to have possible beneficial health effects [67,68]. Beyond isoflavones and soy protein, soy beans are a good source of unsaturated fatty acids, B vitamins, fibre, iron, calcium, zinc and other bioactive compounds that make soy a good candidate for a functional food. Soy fibre content is primarily pectic polysaccharides, a type of vegetal fibre well fermentable by gut microbiota [69]. There are also peptides, such as lunasin (a 43 amino acid peptide) and Bowman-Birk (a 71 amino acid peptide) that are protease inhibitors with negative influence on protein digestion but also with in vitro chemopreventive effect [70,71,72]. Soybean oligosaccharides have been proposed as prebiotic or sugar alternatives [73,74,75].

Soybean contains a wide range of phytochemicals such as phytic acid (1.0–2.2%), sterols (0.23–0.46%) and saponins (0.17–6.16%) with a wide spectrum of potential health benefits [76]. Fewer studies have focused non-isoflavone phytochemicals, such as saponins [77,78,79,80] but with the growing use of soy and soy foods, a more detailed identification and quantification of phytochemicals associated is due [81]. Scarcity of data on non-isoflavone components depends on the great interest that isoflavones aroused for their biological properties.

### 3.1. Protein Content and Protein Quality

One of the concerns of vegetarian diets is the achievement of protein requirements. However, in 2016 the American Dietetic Association (ADA) stated that protein requirements are met if caloric intake reaches recommended levels [82]. Among available plant proteins, soy is the only one that has been shown to have high quality proteins, based on human nitrogen balance studies [83,84,85,86,87,88]. The protein content of soy bean varies between 36% and 46%, depending on cultivars [89,90,91]. Protein quality is an estimation of a single food, often through protein extraction, to obtain information about efficacy in endogenous protein synthesis after the intake. There are several methods developed to define protein quality of foods, mostly based on animal model test, such as Net Protein Utilization (NPU), Nitrogen Balance, Protein Efficiency Ratio (PER), Biological Value (BV) [92] but the most accepted methods nowadays is the Protein Digestibility Corrected for Amino Acid Score (PDCAAS) [93]. Analytical methods for quality protein are summarized in Table 4.

Since 1991, PDCAAS was adopted by World Health Organization (WHO), Food and Agriculture Organization (FAO) and United States Department of Agriculture (USDA), due to the combination of the chemical value of amino acid composition of the test food, with the biological value of true absorbability in its integrity and not only from protein fraction after extraction. For example, BV method implied the use of protein extract, with test animal data and used egg or milk as reference values. PDCAAS uses infant amino acid requirements as reference pattern and digestibility of whole foods from human studies, where available [8,95,96]. The maximum value that can be reached is 100% or 1.00 of PDCAAS, which displays an adequate amount of proteins by a specific food that ensures all the essential amino acids, when the protein intake is provided in appropriate amounts to children aged up to 6 months old (previously at 2 years old but with unsatisfactory information available) and above and adults [8,95]. Even if PDCAAS method was used for a long time, there are few studies on soy. The PDCAAS values of soy have been reported to range from 0.91 to 1.00 [10,93,97,98,99]. Table 5 summarizes data on food protein quality through different methods.

Different amino acid profile and digestibility among soy products depends on fibre and phytate content variability during food processing and these differences may imply small differences in PDCAAS [100]. The accuracy of PDCAAS method has been questioned and it has been suggested that the application of amino acid nitrogen recovery correction factors might mitigate analytical method errors that can represent significant contributors to fluctuation of values, with the consequent improvement in measurement consistency [97]. In a comparison of 3 isolated soy proteins and one soy protein concentrate analysed by two different laboratories, Hughes and co-workers concluded that soy protein had high quality proteins comparable to meat, egg and dairy proteins with a truncated PDCAAS of 1.00 [97]. However, data on true digestibility came from in-vivo rat assay, far from real digestibility during daily human consumption [93].

Protein quality methods have several limitations. Variability between experimental conditions and real habits such as single food or protein isolate intake, absence of antinutrient fractions normally occurring in foods or the effect of other foods in the diet, variability among laboratory procedures, inter or intra variability among test subjects, pre-trial fasting washout phase, limited reliability of faecal losses during non-protein washout, the variable contribution of gut microbiota in nitrogen losses and the use of animal data are common limitations of the analytical procedures [8,92,93,94,95]. To overcome some limitations of method actually used, Digestible Indispensable Amino Acid Score (DIAAS) has been proposed for the qualitative definition of food proteins. DIAAS method does not provide truncated values over 100% and the true digestibility is calculated for single amino acid corrected for ileal and not faecal losses. Moreover, scoring patterns are variable for infants, children or adults with a more precise calibration of this method [96]. At present, the complexity of standardization, especially for human ileal loss data of proteins, does not permit the adoption of this method [101]. Health Canada uses Protein Efficiency Ratio as a standard in evaluation of protein quality [94].

A meta-analysis on nitrogen balance studies, including researches on soy protein sources [83,87,102], demonstrates that there is no significant difference between the intake of vegetal or animal sources to meet nitrogen equilibrium, demonstrating that processed soy proteins are equivalent to animal proteins [99]. Therefore, soy-based vegetable analogues can help to meet protein requirements. 

Soy has been analysed for protein quality and has been found to be very similar to cow milk and egg proteins, traditionally used as standard references [103]. 

In the Framingham Third Generation Study Cohort, link between dietary proteins and musculoskeletal outcomes were investigated prospectively. Appendicular lean mass and quadriceps strength were positively influenced by protein intakes from legume, diary or animal sources [104]. No associations between dietary proteins and bone mineral mass were showed in this protein replete large cohort. In a previous research from the older Framingham Offspring Cohort, quadriceps strength was positively associated with vegetable proteins but not with meat proteins [105]. Soy protein and whey protein equally supported training-induced lean body mass in respect to training-only group; moreover, soy protein but not whey protein, may protect from oxidative damage [106].

In the Italian Levels of Intake Guide for Nutrients and Energy [107], there was no distinction in protein quality for daily requirements. Taking into account the prevalence of vegetable foods in the Italian diet, as recommended, a mean digestibility of dietary protein has been set to 86% [107]. Protein quality data could provide a useful way to define optimal foods to meet protein requirements in low income countries, where food availability can be very limited and the choice of adequate protein sources can be vital, especially in terms of energy-protein interaction [8]. In Western countries, protein needs in vegetarian diets can easily be met, particularly if caloric requirements are achieved [5,92]. The USDA used APP as acronym for “Alternate Protein Product” to identify products with PDCAAS of at least 80% using casein as reference protein, with at least 18% of protein weight per weight (*w*/*w*) in fully hydrated state. Examples of APP are soy flours, soy concentrates and isolates, frequently used in processed meat substitutes (vegetarian patty or burger) [108].

### 3.2. Isoflavones

Isoflavones belong to a functional class of non-steroidal phytochemicals called phytoestrogen (which also include lignans and coumestans) that possess a chemical structure and functions similar to animal endogenous oestrogens [109,110]. The main isoflavones contained in soy beans are genistein, daidzein and glycitein. Plants synthetize isoflavones by various stimuli of environmental stresses, such as infections or paucity of nutrients [111]. Inhibition of pathogens (phytoalexin activity) and molecular signal in symbiotic interactions (in mycorrhizia and rhizobia) are other areas in which isoflavones play an important role [112,113,114,115,116]. Isoflavones are contained in different legumes, such as soy, kidney beans, navy beans, red clover and Japanese arrowroot called kudzu but only soy beans represent a relevant source [117]. Isoflavones content in soy foods is variable among brands and preparations [15,64,118]. Table 6 displays isoflavone content in selected soy foods.

In Canada, isoflavones in soy beans vary from 360 to 2241 mg per kg [119]. Isoflavones content in soy beans from Romania ranged between 210 and 1340 g per kg [120], 1176–3309 mg per kg in USA [121] and 525–986 mg per kg in India [122]. Temperature and soil moisture are crucial for differential synthesis of soy isoflavones during plant growth, with the highest isoflavone concentrations occurring at low temperatures and high soil moisture, mostly depending by the former [63].

Isoflavones have the ability to interact with oestrogen receptors (ERs) due to structural similarity to 17β-estradiol [123]. However, isoflavones have a low estrogenic potency compared to estradiol [124]; indeed, the affinity of soy isoflavones for ERα and ERβ were 1/1000 times and 1/3 times, respectively [125]. The affinity for ERs is higher for genistein than daidzein [125,126,127].

It has been suggested that soy isoflavones may act as selective tissue estrogenic activity regulators (STEAR) and selective oestrogen receptor modulator (SERM), with different mechanisms than direct interaction with receptor [128,129,130,131]. Phytoestrogens produce estrogenic, anti-estrogenic or no activity depending upon the tissue [132,133]. ERα and ERβ display distinct expression patterns in male and female, thus phytoestrogens do not exert their activity as classical oestrogen agonists [134]. Moreover, as a polyphenol subclass, soy isoflavones may have potential antioxidant properties, an essential function in plant management of oxidative stress during photosynthesis [135].

Isoflavones in soy occur mainly in glycoside form, bound to a sugar molecule [33]. Food fermentation during processing or gut digestion breaks glycoside bond by β-glucosidase enzymes of starter microorganisms used in food transformations or by microbial strains of gut microbiota after ingestion. Breaking of glycoside bond leaves isoflavones in aglycone form [33,136,137,138,139]. Dietary habits may have a rapid and strong effect on gut microbiota composition [140,141]. Influence of diet on microbiota is not only limited to fibre, starch, sugar and carbohydrates intake but also involve protein and fat components of foods [142,143,144]. Aglycones occur mostly in the form of genistein (5,7,4′-tihydroxyIisoflavone), daidzein (7,4′-dihydoxyisoflavone) and glycitein (7,4′-dihydroxy-6-methoxyisoflavone) in a ratio of 58:37:5, respectively [18].

**Figure 1:**
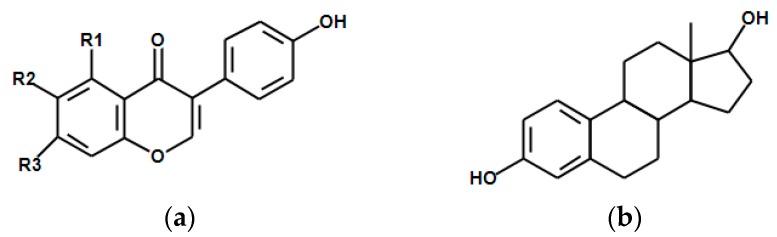

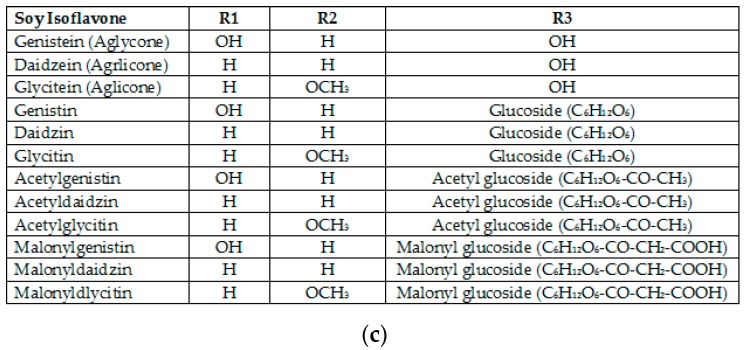
Structure similarity between isoflavones and endogenous oestrogens: (**a**) Isoflavones chemical structure; (**b**) 17 β-estradiol chemical structure; (**c**) Residue substitutions in isoflavone structure.

Malonyl glycoside isoforms are predominant among soy foods [145,146]; however, a minor fraction of isoflavones occurs in aglycone form (especially in fermented products) or acetylglucosides, mainly after thermal treatment [147,148]. The isoforms of soy isoflavones and similarity between isoflavones and endogenous oestrogens are shown in Figure 1.

The presence of aglycone form in food matrix could be responsible for isoflavones detectable in plasma after only 30 min from soy intake and with a peak at 1 h post-meal [149]. During the first hour of digestion, the hydrolysis of isoflavones takes place in the duodenum and urinary excretion occurs within the first 24 h after the soy meal [150]. Hydrolysis in aglycone forms allows passive diffusion and absorption in the upper small intestine; conversely, glycosides are poorly absorbed due to their hydrophilicity [149,151]. After ingestion, aglycone reaches a peak of plasma concentrations after 4–7 h but it takes 8–11 h when glycosides form is ingested [152].

It seems that only 50–60% of Asian people as well as Western individuals among vegetarians can produce 7-Hydroxy-3-(4′-hydroxyphenyl)-chroman (equol) [153,154,155], a metabolite of daidzein detected in urine after the consumption of soy foods; this suggests an inter-individual capacity in the metabolization of isoflavones that may vary the individual effects [156]. Moreover, only 30% of the Western population has been shown to excrete equol after soy meals [154,156]. Equol formation occurs in the distal intestine and colon [157], indicating that the most of soy isoflavone metabolism takes place in human gut by heterogenic pool of different strains of microorganisms that convert glycoside to equol through the intermediate aglycone [150,158,159]. Also intake of other dietary components may influence equol production: diet with high total carbohydrate and low saturated fatty acid has been associated with equol production [160,161] while antibiotic treatments had a negative impact on urine equol excretion [162]. There is a wide inter-individual variation in the pharmacokinetics after soy ingestion of about 15-fold and 12-fold for daidzein and genistein excretion, respectively, with a stronger inter-individual variation up to 600 fold for the equol excretion [160,163]. Different ability of producing equol among people could be an important point of interpretation of discrepancies among soy health effects [156]. This conversion takes place in the gut by the microflora [158,159]. It is plausible that dietary habits which favour specific microbiota population growth could be critical for the isoflavone absorption and subsequent metabolic action [154]. Moreover, soy contains soluble dietary fibre known to be able to act as prebiotic and so the habit of regular consuming soy foods could enhance ability of producing equol [69,73,74], that can explain the more pronounced competence among soy consumers such as Asian people and vegetarians [155]. In-vitro studies suggest that absorbed genistein and daidzein undergo hydroxylation catalysed by P450 enzymes [164,165]. In a second phase metabolism, isoflavones are conjugates with glucuronic acid or sulphate acid by UDP-glucoronosyl transferase or sulfotransferase enzymes in the liver or in the intestinal epithelium [166,167]. The tissue concentration of dietary isoflavones is poorly understood. However, it has been suggested that prostate and breast tissues may have genistein and daidzein concentrations comparable to plasma concentrations, while equol concentration has been shown to be higher [168,169,170,171].

### 3.3. Lipids and Phytosterols

Soy beans are rich in protein but also in unsaturated fatty acid, especially in linoleic acid, a ω6 polyunsaturated fatty acid thought to be beneficial for human health [65]. Among other pulses, soy beans are the only legume providing considerable amounts of α-linolenic acid, an essential ω3 fatty acid [76]. Oil matter is composed by 54% of linoleic acid, 24% of oleic acid, 11% of palmitic acid, 1–9% of α-linolenic acid with a total saturated fatty acid fraction of 9–22% [172,173,174]. The substitution of foods rich in saturated fatty acid with soy foods showed improvement in cholesterol concentration and reduced the risk for coronary heart disease (CHD) [175,176]. Polyunsaturated fatty acid could contribute to the protective effects of soy consumption through the influence on inflammatory parameters [177,178].

Sterols are found in the unsaponifiable matter of soybean fats [179,180]. Phytosterols are steroid alcohols with chemical structures similar to animal cholesterol but with some different carbon substituents and saturations in the side chain [181,182]. Unlike zoosterols, phytosterols are numerous, of which the most abundant are sitosterol, compesterol and stigmasterol [183]. Compared to the good absorption of cholesterol, phytosterols have been considered as poorly absorbable, as reported using radioactive sitosterol with a retention of 0.6–7.5% [184]. Cholesterol-lowering effect of soy phytosterols has been reported since 1958 [185] and more recently extensively demonstrated in humans [186,187,188,189,190,191,192].

The inability of phytosterols absorption suggested a main luminal intestinal mechanisms of interaction for their beneficial effect but these are poorly understood [188,193]. It seems that for the cholesterol-lowering effect, the concurrent presence of cholesterol and phytosterols in the gut is not required [189]. Recently, human studies also suggest anti-cancer properties and improvements in chronic diseases [194,195]. In a cross-sectional study on an Adventist cohort, mean total phytosterol intake was lowest among non-vegetarians and highest among vegan with no differences in plasma plant sterols and cholesterol [196].

### 3.4. Other Bioactive Compounds

Accumulating evidence have suggested that non-isoflavone compounds in soy beans, such as phytic acid and saponins, display a wide range of bioactivities including antioxidative, antiviral, anticancer, hepatoprotective and cardiovascular protective effects [197]. Moreover, some phytochemicals in soy foods may be in higher concentrations than isoflavones, considering mean composition of 0.1–0.3% of isoflavones [198], 0,02% of lignans [199], 1.0–2.2% of phytic acid [200], 0.23–0.46% of sterols [181] and 0.17–6.16% of saponins [201]. It is important to not underestimate the need of established absorption and metabolization of these substances for the achievement of biological effects, depending on food-processing and inter-individual characteristic, especially for the interaction with bile salts in enterohepatic circulation [202]. Future research may focus on the effect on humans of over 100 substances occurring in soy beans, despite the great historical interest for isoflavones [81]. At present, data are scarce and mainly on animals but human interventional studies are needed to understand unresolved mechanisms of action for the soy benefits [81].

Soy protein isolate (SPI) analysis via LC-MS/MS revealed up to 22 saponins [81]. These compounds showed several bioactivities such as protection against cell proliferation and cholesterol reduction [78,80,203]. As for isoflavones, soy foods are the primary sources of saponins [204], which naturally take place as different isoforms of glycosides [205]. Soyasapogenols, aglycones of soyasaponins, do not naturally exist in soy beans, although they may occur in soy products after hydrolysis during food processing [76]. Furthermore, cecal microflora hydrolyse soyasaponins with the break of sugar bond [206]. In human studies, metabolites of soy saponins in 24 h urine collection were not detected but soyapogenols were collected in faecal samples during five days collection, showing that human intestinal microbiota metabolized saponins but with low gut absorbability [207,208]. Biological activities of saponins have been summarized in a detailed review [209]. However, it is noteworthy to underline that in vitro possible effects on health may not be replicated in vivo due to the scarce absorption of these compounds. Among the most recognized, saponins has been shown to have an effect on serum cholesterol, even though absorption does not take place [210,211,212]. In fact, insoluble formation of complexes with cholesterol in the intestinal lumen may inhibit reabsorption of bile salts during enterohepatic circulation for the endogenous and exogenous cholesterol reabsorption after soyasaponins intake [213]. Measurements of saponins in soy-based formulas have been investigated only in few studies [214,215].

Lignans are considered as another group of phytoestrogens in pulses, cereals, seeds and soy beans [117,199]. Bioavailability of lignans has been extensively studied in humans but there is no sufficient data on soy lignans [216]. Intestinal bacteria convert plant lignans to enterolignans, known as enterodiol and enterolactone, that are efficiently absorbed [217,218]. Lignans may exert anticancer activity mediated by interaction with oestrogen receptor; however, antiestrogenic and estrogenic activity of enterolignans in humans are still debatable [219].

Phytic acid and its salt phytate, is a polyphosphorylated carbohydrate widely and naturally occurring in plants. Phytate functions as the storage of minerals and in particular phosphorus, containing about 75% of total phosphorus of the kernel [200]. In soy beans, phytate is the major source of phosphorus but it is also considered a strong chelator for mineral, including calcium, iron and zinc [220]. However, the contribution of phytate antinutrient effect to malnutrition occurs only with high intake of uncooked and unrefined vegetables in association with low micronutrient intake [221]. 

Instead, soy could be a good source of calcium and iron (see Table 1), which is of major interest in vegetarian nutrition [82]. In the past, iron availability from soy beans may have been underestimated [222]. Moreover, recent findings revaluated the notion of the absorption of inorganic iron in respect to eme-iron, thanks to evidence of a more bioavailable plant ferritin in legumes, including soy beans, that could stimulate future biotechnology approaches [223], even though the presence of plant ferritin in soy beans could be very variable [224].

Plasma concentrations of phytate have been directly linked to the intakes, even if human bioavailability studies are very limited [221]. Although phytate is proposed to provide different human health benefits such as antioxidant activity, immune system enhancing, inhibition of pathological calcification, kidney stone formation inhibition, serum cholesterol-lowering effect and the reduction of pathological platelet activity, cancer prevention has been the most debated [225].

Soy beans are also good sources of calcium thanks to the high bioavailability, as defined with fractional calcium absorption of 0.414 and 0.310 from low and high-phytate soy beans, respectively, compared to 0.377 from cow milk [226].

## 4. Health Effects

Isoflavones and soy proteins are the soy nutrients that aroused the most research interest [65,67,227,228]. In 1999 Food and Drug Administration of America (FDA) authorized a health claim about the reduction of coronary heart disease linked to soy protein consumption of at least 6.25 g per portion with a total of 25 g per day, that stimulated the interest of food industry for soy [229]. FDA relied on a meta-analysis on the effect of soy protein on serum lipid profile in 38 clinical trials that evidenced a relationship between soy consumption and total cholesterol, LDL and triglyceride blood levels [230]. Afterwards, claims on soy were released in other countries such as Canada, UK, Brazil, Indonesia and Philippines, mostly for 25 g of soy proteins as intervention for cardiovascular protection [228,231,232]. Vegetarians consuming such quantities of soy were very uncommon; however, cholesterol improvement efficacy of vegetarian diet was established [233]. Moreover, in epidemiological study, lowering effect were achieved with far less soy protein intake than FDA threshold [27,234]. In 2000 the Nutrition Committee of the American Heart Association released a statement for healthcare professionals about soy protection effect against CHD [235]. However, European Food Safety Authority (EFSA) in 2012 stated that cause-effect relationship between isolated soy protein and serum LDL concentration reduction was missing [236]. The beneficial effects on human health depends on both protein and isoflavone content of soy beans, even though the interaction with other soy components cannot be excluded. Interestingly, isolate isoflavone seems to have no effect on blood lipid markers in postmenopausal women [237].

Isoflavones can exert health benefits through oestrogen-dependent and independent mechanisms [238,239]. Concerns about the adverse effect of soy consumption were originally based on animal data [240,241,242]. Today we are aware of the difficulty of extrapolating data from in vivo study to human utility in daily application [243]. In murine models, for example, in utero exposition of estradiol were not comparable to humans due to picomolar versus micromolar concentrations, respectively [244]. Monkey differs from human in isoflavone metabolism, with a more efficiency in daidzein to equol conversion [245]. Non-human primates and rodents have a profoundly different isoflavone metabolism [245,246,247,248,249,250,251].

In epidemiological studies, Asian traditional diets rich in phytoestrogens were linked to lower risks of coronary heart disease [230,252,253,254] and there are some evidence on possible benefit in hormone-dependent prostate [255,256,257], colon, breast [258,259,260,261,262] and ovarian cancers [263,264,265], menopausal symptoms [266,267], osteoporosis [268,269,270,271,272], obesity [273], cognitive dysfunctions [274] and overall risk reduction for non-communicable diseases [109,263,264,275]. Though estrogenic potency of isoflavones was weaker in relation to 17β-estradiol [276], circulating isoflavone levels may exceed endogenous estradiol concentrations in subjects with diet rich in soy foods [126,277].

The debate on the potential perturbation on sex hormones network by phytoestrogens specially pertains infants fed soy-based formula [81] and the putative consequences of massive introduction early in life [278,279]. Moreover, dietary soy intake has been associated with increased risk of thyroid disorders [280], bladder cancer incidence [281], dementia [282] and breast cell proliferation [283,284]. Soy use during pregnancy was shown to alter the epigenome in offspring and can have health implications of in utero stimuli [285].

### 4.1. Possible Beneficial Effects

Epidemiological data showed that isoflavone intake could be responsible for the different CVD rate between Asian and Western countries [264,286,287]. There are possible beneficial effects associated with soy consumption, some of which may depend on its phytoestrogen content. Antioxidative effect of flavonoids may protect from cancer development in which free radicals play a well-known role [123]. In vivo and in vitro studies suggested both estrogenic, antiestrogenic and not hormonally dependent mechanisms of action [288,289,290,291,292]. Furthermore, during the digestion of soy in the gut, the release of biologically active soy peptides, such as conglycinin, takes place. These compounds may have a preventive role in cancer, hypertension, hypercholesterolemia, obesity and oxidative stress [293,294,295,296]. Several evidences have been produced to date, suggesting an overall beneficial effect of isoflavones on human health [297,298].

#### 4.1.1. Cardiovascular and Metabolic Protection

The first report of soy protein influence on lipid metabolism was published in 1967 [299]. In 38 controlled clinical trials, the intake of an average of 47 g per day of soy protein showed a 9.3% reduction in total cholesterol, 12.9% in LDL and 10.5% in triglycerides [230]. The substitution of 25 g of typical Western diet protein with 25 of soy protein reduced LDL by 4% [300].

In vitro studies have revealed that genistein enhanced activities of antioxidant enzymes such as catalase, glutathione peroxidase and superoxide dismutase [301]. The antioxidant capacity of isoflavones has been shown to be comparable to Vitamin E [135,301,302]. However, isoflavones undergoes extensive metabolism in liver and gut, so antioxidant capacity could be affected [303]. The antioxidant capacity could manifest on LDL. In fact, after 17 days of dietary supplementation of soy protein rich in isoflavones, lag time of LDL oxidation by copper was prolonged [304]. The cholesterol lowering effect of soy has been used as therapy for several decades [305]. A recent rigorous meta-analysis confirmed the increase of high density lipoprotein (HDL)-cholesterol with the reduction of total and LDL-cholesterol in adults with normal or high plasma cholesterol [306]. However, the overall effect was weaker than previously reported. A recent meta-analysis of randomized controlled trials of the last 10 years showed significant reduction of serum LDL, triglycerides and total cholesterol concentrations with the increase of HDL concentrations, especially in hypercholesterolemic patients [61]. Stronger effect emerged with whole soy and soy foods compared to soy extracts and with no effect for isoflavone supplementation. Weaker effect in North/South America compared to Europe, Asia and Australia, when stratified by location was observed [61].

Multiple mechanisms are involved and include the action of fibre, protein and isoflavone of soy beans, as well as the intake of soy pectins and the improvement of diet by removing saturated fatty acid sources in place of unsaturated fatty acids from soy [300,307]. Meta-analysis also suggest a modest effect in lowering blood pressure by soy proteins [132,308]. In contrast, only limited evidence on the association between isoflavone intake and risk of hypertension is available [309]. Regarding diabetes, some studies showed a marginal to significant association with risk reduction [310,311,312], despite one report showed null results [313].

In a recent meta-analysis of observational studies on consumption of soy and risk of CVD, a significant negative association was shown, especially in case-control studies and for Asian people but results were non-significant in geographic stratification analysis for Western studies (The Netherlands, Italy and USA) or in stratification analysis by gender for males [314]. No link has been observed between isoflavone consumption and risk for CVD with a little protective effect with tofu consumption [314]. In an Asian population the consumption of 6 g or more of soy proteins showed a reduction of ischemic and cerebrovascular evens, as well as total cholesterol and LDL reduction, as showed by prospective observational studies [315].

Soy may improve endothelial function, slowing progression of atherosclerosis in subclinical phase [230,316,317]. Urinary equol has been inversely related with risk of coronary heart disease in women [254]. Mechanistic studies provide a convincing rationale but additional epidemiological studies are needed to strengthen evidence of the protective effect of soy on cardiovascular disease risk factors.

#### 4.1.2. Anticancer Properties

The beneficial effect of soy food in the diet of Asian and Western women has been highlighted, even for those with breast cancer diagnosis [318,319]. In a Chinese cohort of 73,223 women with high breast cancer risk, a strongly statistically significance has been showed in a prospective study between soy food intake and reduced risk of premenopausal cancer risk but without any association with post-menopausal breast cancer [320].

Anticancer effects of soy have been largely ascribed to isoflavones, which can modulate cell cycle, apoptosis, differentiation, proliferation and cell signalling [321,322,323,324].

An inverse association between soy food intake and breast cancer in Asian population has been reported [325]. Even prostate cancer had a lower incidence rate in Asian population and this phenomenon has been linked to intestinal bacteria competence in daidzein to equol conversion [326]. However, while soy use significantly reduced prostate cancer diagnosis, prostate specific antigen levels in serum (PSA) was not affected by short term soy isoflavone intake [327,328,329,330,331].

Genistein inhibited Human DNA Topoisomerase II enzyme activity in vitro, so in high amount could have an anticancer activity [332]. In breast cancer cell lines phytoestrogens modulated chromatin transcription through epigenetic modifications such as methylation and acetylation of histones [333]. In fact, cancer is characterized by gene expression de-regulation [334]. A protective action during the cancer promotion phase may explain health benefits such as reduced breast cancer risk and reduced recurrence in Asian women exposed to soy since infancy [335].

A recent systematic review and meta-analysis about the association between soy isoflavones consumption on colorectal cancer risk, showed a significant lower relative risk, with statistical significance in pooled analysis for soy foods, Asian populations and case-control studies [336].

A systematic review and meta-analysis on association between isoflavones and endometrial cancer show that dietary soy isoflavones from soy beans and soy foods were associated with the reduction of the risk of endometrial cancer (OR: 0.82, 95% CI 0.72 to 0.92), with statistical significance in pooled analysis for geographical regions for both Asian (China, Japan) and non-Asian countries (USA, Sweden, Italy, Australia) [337].

It seems plausible that the best advantage in soy food consumption comes from little but frequent intakes over the day of phytoestrogens-rich foods [145]. 

Risk reduction of breast cancer in case-control studies was associated with higher soy intake, particularly early in life [26,320,338,339].

A recent comprehensive meta-analysis including 143 studies on the association between isoflavone intake and cancer risk distinguished results by study design as only prospective studies are considered valid to draft evidence of association between foods and health outcome: the report showed significant results for the association between isoflavone intake and decreased risk of stomach and lung cancer, while nearly significant decreased risk of breast and colorectal cancer [340]. However, further prospective studies are needed to confirm the latter retrieved association.

#### 4.1.3. Menopause and Osteoporosis

Recent studies have pointed out the importance of nutritional factors on gynaecological-related conditions [341,342]. In particular, the link between soy foods and menopause has been shown by clinical and epidemiological data [343,344]. In Western countries the use of phytoestrogen supplements among postmenopausal women was recently increased as an alternative to hormone replacement therapy [345]. In a position statement of 2015, a consensus of soy isoflavones as a first-line approach to vasomotor issues in menopause has been achieved [346]. Grade I of evidence in efficacy of isoflavones on menopausal hot flushes has been declared in a position paper of the International and Austrian Menopause Society [346].

In 2011, a review of literature including Cochrane Library, conducted meta-analysis of randomized controlled trials and found a reduced resorption turnover through bone markers and bone mineral density in menopausal women using soy isoflavones [347]. 

The reduction of bone mineral density in menopause can be caused by decline in endogenous oestrogens and hormone replacement therapy or soy supplements are able to reduce risk of osteoporosis [18]. Peri-menopausal bone loss was attenuated by consumption of soy protein isolate rich in polyphenols [268]. In a recent randomized crossover trial, soy isoflavones to equol conversion capacity was linked to increased bone calcium content in postmenopausal women [348]. Isoflavones in aglycone form could be crucial for favourable bone effects as revealed by an Italian 3-years intervention study of genistein supplementation with the improvement in bone mineral density [349]. However, some data were inconsistent or with absence of effect in bone formation markers, maybe in part dependent by the usage of isoflavones in glycoside form [350,351,352,353,354,355,356]. Clinical studies have failed to show improvements in calcium balance with soy protein intake in comparison to animal protein [357,358]. In a prospective epidemiologic study on US Seventh-Day Adventists participants, soymilk or diary consumption were associated with similar degree to significantly lower risk of osteoporosis [359].

There is some evidence on the reduction frequency and severity of hot flushes in post-menopausal women by soy foods [266,343,360,361]. Isoflavones intake has been proposed as a safer alternative to hormone treatment in controlling hot flushes [362]. As for other health effects, bioconversion of isoflavones to active metabolites could be crucial for relief from menopausal discomforts [361,363,364].

In addition, soy beans provide high quality protein that could be critical for bone health [365,366]. Furthermore, soy beans are rich in calcium that may contribute to the achievement of the post-menopausal women dietary reference intake of 1200 mg per day [107,367]. Calcium bioavailability from soy was optimal, despite phytic acid and oxalate acid retention in seeds [226,368].

#### 4.1.4. Other Beneficial Effects

There are only limited information on body weight loss in human studies [273,369,370]. Soy is thought to alleviate complications of obesity by decreasing lipoprotein lipase activity and ameliorating insulin resistance [369,371,372]. The limited content of carbohydrates and higher content of protein makes soy a good candidate for management of glycaemic response in diabetes and insulin resistance patients and more in general in metabolic diseases [373,374]. High-protein weight-loss diets with meat or soy-based protein sources showed similar appetite control and weight loss among obese men in a randomized crossover trial [375].

Limited data suggested that soy can be beneficial on renal health [376], cognitive functions [377], immunity [378] and reproduction [379].

As mentioned above, intestinal activation of soy phytochemicals by microbiota could be crucial for absorption and efficacy of soy bioactive compounds. Furthermore, soy itself can shape gut microbiota [380,381]. Soy and soy foods can provide substrates useful in modulating the growth of specific bacterial strains [382]. Soy-based drinks, widely used in Western and Asian countries, can provide polysaccharide and proteinaceous compounds, easily fermentable by gut microbial populations [383,384,385,386]. Influences on gut microbiota could be partially linked to the beneficial effects of soy on metabolic syndrome.

Intakes of 500 mL of soy-based drink in obese or overweight men seemed to change main gut microbial phyla by stimulation of Bacterioidetes and Proteobacteria growth and reduction in relative abundance of Bifidobatera and Firmicutes. Different formulation in protein content can stimulate differential bacterial growth [380]. However, replacement of cow milk-based formula with soy-based formula in feeding infants 3–8 months old, stimulated no variation in selected Bifidobacteria species [387]. Isoflavones are naturally produced by plants as chemical defence from fungal and bacterial infection and, therefore, may provide a contribution in shaping of gut microbiota through antimicrobial activity [388,389]. Also saponins could have a concerted effect with isoflavones on simulation of microbial population that could modulate isoflavone activation by stimulating equol producing bacteria [381,390]. Therefore, it is plausible that isoflavones efficacy depends on inter-individual bioactivation capacity but, at the same time, the presence of soy phytochemicals and isoflavones in gut lumen may act on microbiota activation competence, thereby promoting absorption and efficacy of soy components itself. Traditional production of soy foods, including different fermentation procedures, imply the occurrence of new bioactive chemical substances in food matrix that are relevant in shaping of microbiota (see also section below of soy foods transformation) [391]. The health-promoting effects of fermented soy foods have been shown in different disease [392,393,394]. Unfortunately, most of the data came from animal studies, which are very hard to transfer into human physiology [395,396,397,398,399,400,401,402].

### 4.2. Possible Detrimental Effects

#### 4.2.1. Thyroid Gland Disturbance

In vivo studies on thyroid functions have raised some concerns about perturbation effects of soy in people with clinical or subclinical thyroid diseases [403,404]. However, it is unlikely that soy foods can alter thyroid function in euthyroid individuals even in iodiopenic circumstances [405,406,407]. In vitro inhibition of thyroperoxidase activity was not followed by disturbance of thyroid gland metabolism in people consuming foods rich in flavonoids, even with the intake of isolated genistein in post-menopausal women [408]. In a cross-sectional epidemiological study on North American churchgoers belonging to the Seventh-Day Adventist church, a significant positive association between soy intake and thyroid-stimulating hormone (TSH) in women but not in men was found [409]. Though, the relatively low intake of iodine in the cohort can partially explain the thyroid susceptibility to soy. Soy flour based infant formula was linked to goitre before iodine fortification [410,411,412]. In vitro studies showed an inhibitory effect of soy isoflavones in aglycone form on thyroxine synthesis by thyroidal gland only in absence of iodine [280]. Goitres observed in infant fed with soy-based formula were reversed with iodine fortification [408]. Even if hypothyroidism characteristic is the slow growth, this phenomenon was not seen in infants fed with soy formula. More long term epidemiological studies are needed to verify soy-thyroid interaction in real conditions and to discriminate from clinical trials, which often use isolated isoflavones with a wide range of concentrations and heterogenic conditions [413].

In light of the inhibition effect of soy foods on the absorption of thyroid hormone replacement therapy, it has been suggested that hypothyroid patients should outdistance drug intake from soy consumption and more in general from any food rich in fibres and phytochemicals [414,415].

#### 4.2.2. Sex Hormones Perturbation

The action of phytoestrogens as selective oestrogen receptor modulators, makes it unlikely to have negative effects in oestrogen pathways. Perturbation of sex hormone network and infertility issues attributed to soy foods are in strong contrast with that found in large populations of soy-consuming countries. The link between soy and reproduction has been postulated in 1940 after recognition of endocrine disruption by isoflavones of red clover in pasture given to ewe flocks, resulting in infertility syndrome called “clover disease” [416]. In 1976 Shutt speculated a defence action of some plants with antifertility factors for mammalian predators [417].

Little perturbation on reproductive health have been reported, including feminizing effects, erectile dysfunction and reduced libido linked to very large intakes of isoflavones, even though available data revealed only minor detrimental effects or no impact on health [418,419]. However, no oestrogen perturbations were reported in clinical studies with high exposures to isoflavones in men [420]. In a cross-sectional study, soy foods intake was inversely linked to sperm concentration in 99 men despite no changes in quality parameters and the cohort was enrolled from sub fertile couples group with no detailed information on soy intakes [421]. Conversely, sperm count and motility were positively related to isoflavone intake [422] or with no correlation between isoflavone intake and sexual hormones or semen quality [423]. Testosterone levels were evaluated in a meta-analysis of 32 reports with no conclusive interaction between soy or isoflavone intake and free testosterone concentrations [424]. The majority of information about soy to sex hormones interaction came from in vitro and in vivo studies [379,425].

Regarding reproductive health in women, a cross-sectional study of 11,688 women aged 30–50 years of North American Adventist church showed that high intake of isoflavones was related to increased risk of nulliparity and nulligravity [426]. The effect on pregnancy was observed for intake ≥40 mg per day. A systematic review of literature showed that effect of soy on sex hormones in pre- and post-menopausal women had very small effect, as well as on thyroid function in post-menopausal women [133].

Soy-based formula is commonly used in Western countries for allergy to cow milk proteins or as alternative vegetarian choice [427,428]. Urinary total isoflavones among infants fed with soy formula were 500 times more concentrated compared to cow milk formula fed infants [429]. Even plasma isoflavone concentrations were 10 times more concentrated in soy formula fed infants [430].

In a longitudinal epidemiological study, introduction of soy products into infant diet before 4 months of age was associated with a 25% higher risk of menarche before 12 years of age, in support of mild endocrine disrupting effects of soy isoflavone exposure [431]. However, these preliminary findings did not report the exact amount of soy intake in the sample so it was not possible to assess a true dose-response relationship. A retrospective epidemiological study did not find a link between exposure to soy formula and reproductive outcomes [432]. In a cross-sectional study on Seventh-day Adventist girls with age ranged between 12 and 18 years, no correlation between soy intake and the age of menarche was found [433].

#### 4.2.3. Procancer Activity

Like many other potential detrimental effects, concerns about the interaction between intake of isoflavones from soy and neoplastic events came mainly from in vitro and animal model studies [434,435,436,437].

In the European Prospective Investigation into Cancer and Nutrition Study cohort (EPIC) there were no increase in cancer risk caused by soy isoflavone intake [438].

In 2010 a meta-analysis found no effects on postmenopausal women by isoflavone intake, assessed with mammographic density [439]. Similarly, a blinded randomized clinical trial found no variation in breast MRI fibroglandular tissue density or mammographic density in women with breast cancer and at-risk women with 1 year soy intake intervention [440].

Overall, epidemiologic studies support a protective role of soy foods in breast cancer, as confirmed by meta-analysis of cohort and case-control studies in Asian and Western countries, with pre- and post-menopausal women and with a more protective effect in Asian Women [441]. A large prospective epidemiologic studies showed that soy intake after diagnosis of breast cancer favourably affected prognosis [318]. British women in the EPIC-Oxford arm, which oversampled vegetarians, had isoflavone intake similar to Asian people in epidemiologic studies. Nevertheless, high-soy consumption in this cohort was unrelated to breast cancer risk, possibly due to adoption of dietary habit only in adult age and with low soy consumption in infancy [442]. The “early-intake” hypothesis could explain discrepancies in epidemiologic data from different countries [443].

## 5. Soy Foods

There are several soy foods, traditional Asian foods (fermented or not) and new generation products, such as soy milk, edamame, tofu, soy cheese, miso, soy sauce (tamari, shoyu), tempeh, natto, sufu, yuba, soy flour, soy protein isolated (SPI) and meat analogues also called Textured Vegetable Protein (TVP). Table 7 shows a description of selected soy foods.

In European countries, there is a wide variation of soy foods consumption. In Germany, Spain and The Netherlands, most frequently consumed has been reported to be beans and sprouts. In Italy, grain products were the most frequently consumed soy foods. In Denmark, soy meat substitutes were the most popular, instead; in the UK soy dairy substitutes were the main soy-based food consumed [43]. TVP provides a good source of protein in the form of soy meat analogues, readily digestible. In fact, protein digestibility was 66.1% and 63.4% for TVP defatted soy four and TVP protein concentrate, respectively, in comparison to 73.2% for beef [444].

Food processing can dramatically perturb the composition of soy products by altering nutrient and antinutrient content; moreover, artificial compounds from processing may also occur [445,446]. For example, processing of soy beans may improve nutritional quality by the reduction of antinutrient naturally present in foods but Maillard reactions may reduce amino acid bioavailability [447,448]. Heat induced interaction between amino acids and sugars results in the formation of Amadori’s compounds of Maillard reaction consisting in browning products and acrylamide [449]. Highly processed soy foods can lose up to 80% of their isoflavone content [33].

Numerous aromatic compounds were isolated from miso and soy sauce [450,451]. Furthermore, a 1.7 fold increase in folate content was reported after soy bean fermentation in tempeh [452]. The fermentation process of soy foods not only affects sensory properties and shelf life but also changes in nutritional value and digestibility may occur [453]. Noteworthy, food processing may influence the final phytoestrogen content [454]. Also starter microorganisms used for fermentation could give additional beneficial human health properties such as probiotic functions [455].

In countries of European Union, definition of milk is “substances secreted from mammary gland” [456]. For non-compliance reasons, “soy milk” definition is not permitted for sale. 

Even though soy based formula was often used for infants with atopic disease, 10–14% of subjects with a diagnosis of cow milk allergy were also positive for the soy allergy [427,457,458,459]. Soy based formula is preferred by parents with vegetarian lifestyle [427].

Soy-based drink full-fat contains about 24% of calories from protein (mainly glycinin and conglycinin), 45% from carbohydrate, with absorbable and not absorbable mono and polysaccharides, including fructose, glucose, galactose, sucrose, stachyose, raffinose and others and 31% of calories from oil [385,386].

Soy beans, SPI, tofu, soy milk and fermented food products such as miso, natto, tempeh and soy sauce are the most considerable dietary source of isoflavones in human nutrition [460,461]. Different processing method could be responsible for isoflavone isoforms content in soy-based formulas [214].

In soy flour, total isoflavone concentrations range from 60 to 265 mg per 100 g, tofu may contain between 5.1 and 64 mg per 100 g of total isoflavones, soy milk 1.3–21 mg per 100 g, tempeh 6.9–63 mg per 100 g, soy sauce 0.1–2.3 mg per 100 g [64], miso 23–126 mg per 100 g [64,462] and natto 20–124 mg per 100 g [463,464].

Deep-frying of tempeh reduced isoflavone content by almost 45% [465]. Temperature is critical for retention of isoflavones: in fact, soymilk film formed during boiling, called Foo joke or Yuba, has been reported to have 196.05 mg per 100 g of isoflavone compared to 44.67 mg per 100 g after cooking [64]. Also soaking temperature and time in edamame production (immature soybean seeds), contributes to isoflavone variation [466].

Bioavailability of isoflavone isoforms is still a debate. Soy drinks and TPV contain predominantly glycoside compounds, while tempeh, miso, natto and fermented soy milk (including sufu) contain aglycone forms. Aglycones are more readily absorbed than glycosides and liquid matrix enhances absorbability compared to solid foods [467,468,469,470].

### New Generation Foods

The nutritional value of soy foods obtained from SPI could be lower than unprocessed or minimally processed foods [471]. Frequently, meat analogues are prepared by extrusion with different moisture conditions, which confer meaty consistency, much more acceptable for consumers, thanks to re-texturization through formation of disulphide bonds [472,473]. Change in molecular structure through formation of disulphide bonds plays a critical role in texturizing of soy fibrous protein in meat analogues [474]. However, non-covalent interactions and disulphide bonds formation during cooking and extrusion may lead to changes in the quality of proteins and nutritional value of products through Maillard reaction, gelatinization and autoxidation [444,475]. Studies which have investigated the worsening of nutritional characteristics of extruded products were carried out under low or moderate moisture conditions. On the contrary, working at high moisture and low temperature during extrusion process could be useful to preserve nutritional value [472,473]. Sometimes, digestibility of texturized soy foods has been found to be higher than unmodified proteins [476]. No differences in isoflavone patterns between unextruded and extruded soy meals were observed but bioavailability was not be tested [477].

Meat analogues have higher polyunsaturated fatty acids, potassium, calcium and phosphorous compared to ground beef, with no alteration of amino acid profile and biological value after home cooking [478]. New generation soy foods resulted from recent advances in improving taste characteristics through plant breeding and new processing technologies [479,480].

Soybean oil is widely produced and consumed as cooking oil worldwide [481]. Soy oil have negligible amounts of isoflavones because it is often obtained by alcohol extraction, which is the same procedure used for soy defatting. Aqueous extraction can obtain high isoflavone concentrations due to the hydrophilicity of soy isoflavones [15]; these compounds are tightly associated with proteinaceous matter and food processing such as alcohol extraction significantly decreases phytochemicals content [371].

Variability of isoflavone content was detected in vegetarian food alternatives [482]. The most refined soy foods can lose up to 80–90% of isoflavone content during processing [33,483,484]. Soy burgers have 0.1–26 mg per 100 g of total aglycone equivalents, soy yogurts 1.6–11.8 mg per 100 g [22,485,486], soy drinks 1.0–21 mg per 100 g [64,485] and soy cheeses 2.3–33 mg per 100 g [22,486,487]. Usually, bakery soy-based products retain low concentration of isoflavones [64,486,488,489]. Soy milk formulas may contain up to 31 mg per 100 g [64].

Overall, the low concentration of isoflavones does not qualify the new generation soy foods as good sources of phytoestrogens, effective for the reduction of chronic disease risks, nor for alleged detrimental effects for soy intake [490]. However, aqueous extraction method used for SPI production could enhance aglycone retention due to β-glucosidase activity resulting from processing below 50 °C [454]. Differences in isoflavone content may depend on distribution of isoflavone forms, instead of absolute content, as isoflavones seemed to be very stable at different temperature and pH values [445,454,491]. Even so, processing may affect nutraceutical values of soy and soy foods by altering retention and distribution of bioactive substances such as isoflavones [22,492,493,494].

Interconversion among different isoflavone isoforms could explain the heterogeneity in phytoestrogenic efficacy from various soy foods [495,496]. Indeed, thermal processing decreased malonyl glucosides and increase β-glucosides and aglycones [497]. Deeply processed soy foods could have very limited concentration of isoflavones due to solvent extraction that limits phytochemicals retention [371,446,454]. Conversely, soaking and fermentation processes, typical of traditional Asian soy foods such as tempeh, soy sauce, miso, natto and traditional tofu, could help bioavailability of isoflavones thanks to the increasing of aglycone form concentrations [461,466,467,468,469,470].

## 6. Food Safety

The use of soy in Japanese and Chinese diets has a traditional usage history of safety that was recently extended to Western countries [121]. However, some studies raised concerns regarding soy foods use, such as tofu, on cognitive outcomes [282,498,499]. In a short term dietary intervention study, high soy diet versus low soy diet have no adverse effect on cognitive function and mood in healthy young students [500]. Achievement of some level of college education was non different between soy or cow milk formula fed in infancy [432].

Daily administration of 54 mg of genistein in aglycone form to menopausal women for 3 years did not affect thyroid function [406]. Moreover, 200 mg of isoflavones daily administration for 2 years did not influence TSH [501]. Isoflavones showed an overall good profile of safety for thyroid function [413].

In 2015 an EFSA panel concluded that the intake of 35–150 mg per day of isoflavones from supplements or foods does not have adverse effect on sex hormones-responsive tissues such as breast and uterus or thyroidal gland up to 2.5 years duration of intake [502].

Clinical and prospective epidemiological data show safety of use during isoflavone exposure in women [503,504]. In a double blind randomized intervention study of 12 months, soy intake did not worsen breast fibroglandular tissue density in breast cancer patients previously exposed to antineoplastic treatments or in women at high risk [440]. Moreover, exposure to isoflavones or soy protein in breast cancer patients was associated with reduced mortality and cancer recurrence in women with ER(+) and ER(−) breast cancers [318,349]. No contraindication for isoflavone intake in women under treatment with tamoxifen or anastrozole has been observed [505,506,507], rather, soy consumption enhanced efficacy of anticancer treatments [508,509].

### 6.1. The Early Stage of Infant Nutrition

Infant feeding with soy-based formulas could be a leading cause of concerns for soy use. Early and massive exposure to soy bean bioactive compounds, concurrently with low body weight, raised doubts about the perturbing influences in sensitive time windows of development during infancy. Soy formula was used worldwide over 60 years to feed millions of infants with no observable adverse effects [427]. First soy-based infant formula was reported over 100 years ago [510]. In the past, Italian infants fed with soy flour based formulas had a poor response to polio vaccine [511,512,513]. However, modern soy formulas showed good immunodevelopment response to anti-polio [514]. Limitations of soy formula based on soy flour gave rise to SPI formulas with a better nutritional profile in PDCAAS and reduced non protein ingredients such as protease inhibitors, fibres and phytate [515]. In the National Health and Nutrition Examination Survey 2003–2010 (NAHNES 2003–2010), among infants 0 to 12 years old consuming formulas or milk, 12% were fed with soy formula [516]. Even though it must be stated that human milk is the best nutrition choice for infants [517], in USA and in European Union Countries, infant soy formula SPI based are considered as safe as cow milk based infant formula [515,518]. This statement is not universally shared: in fact, Australian College of Paediatrics claimed that soy based formula is not equivalent to milk formula in micro and macronutrients and infants fed with soy formula need more energy [519]. EFSA released formulation guides for infant formulas, including soy formula, in relation to the needs of infants [518]. Despite the possibility that phytates content of soy-based formula may inhibit micronutrients absorption, no difference was observed in growth and micronutrients status, including iron, of children fed with soy formula compared to cow milk formula [432,515,520,521,522]. Moreover, bone mineralization was at least equal to infants fed with cow milk formula or human milk [523,524,525]. Even if circulating plasma isoflavones were from 13,000 to 22,000 times higher than estradiol in early life of soy-fed infants [430], plasma isoflavones do not seem to be linked with oestrogen status [526]. However, 99% of circulating isoflavones exists as sulphate or glucuronide conjugated forms in biologically inactive state, as results of first-pass metabolism via gastrointestinal absorption and liver metabolism [527]. No long term effects on sexual development and maturation have been shown in soy formula fed infants [432,528]. Moreover, soy infant formula milk may promote bone development and support normal growth [529,530,531,532]. Early exposure to soy formula and soy foods may provide health benefits in body composition and in prevention of some cancers [531,532,533].

Inter-individual absorption and metabolization processes of soy bioactive substances are still poorly understood. No difference in absorption and metabolization rates was found after measurement of daily urinary isoflavones excretion of 2–16 weeks old infants in relation to adult samplings [534]. Conversely, another study found that infant absorption under 4 months old was negligible, probably due to the gut microbiota unable to process isoflavones [535]. Additional evidence showed that a 20 g roasted soy beans intake by lactating woman, significantly increased isoflavone detection in breast milk [536]. A controlled randomized interventional pilot study showed that term breast milk of 18 mothers consuming 250 mL soy drink with 12 mg of isoflavones, contained about 3 μg of isoflavones per litre [537]. One serving of soy beverage intake for 2–4 days increased 10–15 times breast milk isoflavone content in lactating women [538]. These quantities were lower than the concentrations of isoflavones found in soy formulas but it should be investigated if the intake through mammary gland secretion after paracellular transport into the alveoli could provide a more bioavailable form of isoflavones.

### 6.2. Soy Allergy

Soy, milk, peanuts, wheat and eggs account for about 90% of allergy in atopic children, with the latter the most frequent and severe [539,540]. Even though studies with clinically confirmed investigation are scarce, prevalence of soy allergy appears to be low with rare anaphylaxis reactions to soy containing foods.

Frequently soy formulas were prescribed for adverse reaction to cow milk, however, a soybean glycinin protein of 30 kDa cross-reacts with casein [541]. Enterocolitis and other clinical manifestations of soy allergy are overlapping to those of cow milk allergy [542].

Self-reported soy allergy in USA ranged between 0.1% and 2.7%, with higher prevalence among children up to three years old [543,544,545]. In Europe, the highest prevalence (1.2%) was reported among Swedish children of four to eight years old [546,547]. Generally, self-reported allergy at all ages in other countries of Europe were ≤0.6 % [548,549,550,551,552].

Fermentation of soybeans and soy foods reduces allergenicity, showing very low immunoreactivity of yogurt followed by miso and tempeh [553] but not for soy sauce [554].

Soy allergy does not seem to be an issue in human nutrition, even in infant, taking into account the highest prevalence in children of self-reported cow milk allergy of 10.8% in Iceland [551] and with the lowest of 1.3% in Norway [555]. Moreover, the most recent approach to infant nutrition contemplates to not delaying the introduction of any food beyond 6 months of age, even though potential allergenic, to prevent allergy [556], also from peanuts, which causes the most severe food allergy [557].

## 7. Conclusions

Soy consumers are generally driven to find alternative protein sources from animal ones because of a vegetarian style adoption [53]; however, concerns for soy safety in breast cancer and thyroid influence have been reported [558].

The adoption of vegetarian diets has increased over the last decades together with the availability of soy surrogates from grocery stores. The acceptance of milk and meat analogues have already aroused the development of alternative production processes to obtain more safe and nutritionally adequate products [559]. Acceptance of vegetarian options by middle school students seems to pose no complications in substitution of meat based meals with soy-based ones and could facilitate the meeting of saturated fat limit intake in school meals [560,561,562].

Inter-individual variation of isoflavone activation capacity (mainly located in to the microbiota), differences in cultivar, environmental factor, culture methods, storage, food processing and other confounders (i.e., other dietary habits), pose challenges to forecast the bioavailability and pharmacokinetics of phytoestrogens [121,563,564]. It should be noted that the composition of soy food may differ substantially from unprocessed soy beans. More data on safety are needed but soy foods seems to have a favourable effect on women health, in particular if gut daidzein to equol conversion readily occurs [565]. Soy food can be safe and even beneficial if consumed by women with breast cancer diagnosis [566,567,568]. Among soy products there are remarkable differences in nutrients and phytochemicals composition that need careful interpretation of data on soy effect from different soy foods [569].

Sex hormone network and thyroid gland perturbation seems to be unlikely, especially with low isoflavone intakes actually reported in vegetarian. Overall, the low content of bioactive compounds in second generation soy foods and moderate amounts in traditional soy preparations offer modest health benefits with very limited risk for potential adverse health effects [570]. At the same time, to have the beneficial effects of soy isoflavones, intake should be at least of 60–100 mg per day [571], at present not easily reached in Western countries.

The “early intake” hypothesis for soy could explain discrepancies in epidemiological data on soy consumption and benefit for human health, especially in breast cancer risk reduction [572]. However, soy and soy food intakes are growing in Western countries, with particular contribution of vegetarians who could harvest the soy benefits. The labelling of soy foods with isoflavones concentrations could be a helpful information for consumer, along with shared guidelines for soy intake in order to obtain health benefits and with the warnings for upper level of intake, especially for long term consumption.

In addition, more clinical trials on other soy bioactive substances could clarify single components/phytocomplex efficacy and safety, even if many of these compounds are recurring in other vegetal of the same botanical family [573].

Despite the high intakes in Asian countries, there is still a paucity of long term data on soy consumption in Western countries. Currently, the use of soy foods in infancy, including soy-based infant formulas, was not linked to adverse events. However, absence of evidence is not an evidence of absence. With the growing adoption of vegetarian diets and with the choice of soy formulas by Western vegetarian parents, a broader characterization of the role of soy foods in Western nutrition could help the acceptance of plant-based new generation foods.

The transfer of data from in vitro and animal model to human health, as well as clinical trial results with isolate or concentrate fractions from soy [574], as well diverse nutraceuticals supplements [575,576,577,578,579,580,581,582], must be carefully interpreted. The disturbing effect of soy on thyroid hormones in subclinical hypothyroidism has been debated but it seems to be a mere goitrogenic mechanism, so it is crucial to satisfy adequate intakes of iodine from foods or by fortification, especially in vegetarians [583,584,585,586,587,588,589]. On the other hand, there are other promising foods, such as lupins, mushrooms or single cell proteins (often mycoproteins), as candidates for meat substitutes production. The use of various protein sources could be useful for avoiding a monochromatic nutrition in vegetarians, widely based on soy [13]. Key nutrients in soy (such as proteins, minerals, vitamins and phytochemicals) are also shared by others plant foods, so it is unnecessary in vegetarian diet to always choose soy and soy foods. Seeds, nuts, cereals and other pulses can be good sources of protein that can be alternatively chosen to obtain a nutritionally adequate and healthy diet, as proposed in VegPlate method [590] and the use of soy can be limited to personal taste and habits.

## Figures and Tables

**Table 1:** Vegetable protein sources from legumes ^1^.

Food	Energy ^2^	Protein ^3^	Carbohydrate ^3^	Fats ^3^	Fibre ^3^	PUFA ^3,4^	Iron ^5^	Calcium ^5^
Azuki beans	329	19.87	62.90	0.53	12.7	0.113	4.98	66
Fava beans	341	26.12	58.29	1.53	25.0	0.627	6.70	103
Chickpeas	378	20.47	62.95	6.04	12.2	2.731	4.31	57
Green peas	352	23.82	63.74	1.16	25.5	0.495	4.82	37
Kidney beans	333	23.58	60.01	0.83	24.9	0.457	8.20	143
Lentils	352	24.63	63.35	1.06	10.7	0.526	6.51	35
Lima beans	338	21.46	36.38	0.69	19.0	0.309	7.51	81
Lupins	371	36.17	40.37	9.74	18.9	2.439	4.36	176
Mug beans	347	23.86	62.62	1.15	16.3	0.384	6.74	132
Mungo beans	341	25.21	58.99	1.64	18.3	1.071	7.51	138
Navy beans	337	22.33	60.75	1.50	15.3	0.873	5.49	147
Peanuts	567	25.8	16.13	49.24	8.5	15.558	4.58	92
Pinto beans	347	21.42	62.55	1.23	15.5	0.407	5.07	113
Soy beans	446	36.49	30.16	19.94	9.3	11.255	15.70	277

^1^ From USDA Food Composition Databases [15]. ^2^ Kcal per 100 g. ^3^ g per 100 g. ^4^ PUFA: polyunsaturated fatty acids. ^5^ mg per 100 g.

**Table 2:** Soy consumption per day ^1^.

Nationality	Soy and Soy Foods ^2^	Soy Protein ^2^	Isoflavones ^3^
USA	NA	NA	0.73–3.3
Europe	NA	NA	0.37–4.5
Vegetarians and soy users	NA	8.42–9.25	3.2–30
China	23.5–135.4	2.5–10.3	6.2–75.7
Japan	50.7–102.1	6–11.3	22.6–54.3
Korea	21.07	7.4–8.5	14.88

^1^ Adapted from references [22,25,31,37,43,52]. ^2^ g. ^3^ milligrams. NA: Not available.

**Table 3:** Isoflavone content in soybean from different cultivation countries ^1^.

Country	Isoflavone per 100 g
Australia	120.84
Brazil	99.82
China	118.28
Europe	103.56
Japan	130.56
Korea	178.81
Taiwan	85.68
USA	159.98

^1^ From USDA Database for the Isoflavone Content of Selected Foods [64].

**Table 4:** Analytical protein quality methods ^1^.

Method	Description
Net Protein Utilization (NPU)	Difference between nitrogen retention in carcass of animal group feed with test protein and nitrogen content in carcass of animal group with free protein diet normalized for dietary nitrogen intake.
Protein Efficiency Ratio (PER)	Gain in body mass of animal model divided for protein intake
Nitrogen Balance	Protein intake requirement to attain nitrogen equilibrium. The difference between nitrogen intake and nitrogen loss with urine and faeces (nitrogen absorption and retention).
Biological Value (BV)	Nitrogen absorbed form test protein divided for nitrogen incorporated into the body and standardized for a reference protein.
True Digestibility	Difference between nitrogen intake and nitrogen loss corrected for protein-free diet loss.
Amino Acid Score	mg of amino acid in 1 g of test protein divided for mg of amino acid in requirement pattern.
Protein Digestibility corrected for amino acid score (PDCAAS)	Amino acid score multiplied for true faecal protein digestibility.
Digestible Indispensable Amino Acid Score (DIAAS)	Amino acid score for selected pattern multiplied for true ileal amino acid digestibility.

^1^ From references [8,92,93,94,95,96].

**Table 5:** Protein quality through different methods ^1^.

Source	PDCAAS	Digestibility (%)	Amino Acid Score	PER	BV
Soy	0.92–1.00	95–98	0.94	2.2	74
Wheat	0.25	96–99	0.26	0.8	64
Beef	0.92	94–98	0.94	2.9	80
Egg	1.00	97–98	1.21	3.8–3.9	100
Milk	1.00	95	1.27	2.5–3.1	91

^1^ From references [8,10,93,97,98].

**Table 6:** Isoflavone content in soy foods ^1^.

Food	mg per 100 g
Miso	41.45
Edamame	17.92
Natto	82.29
Soy cheese ^2^	6.02–25.72
Soy four, textured	172.55
Soy four, defatted	150.94
Soy lecithin	15.7
Soy protein concentrate ^3^	94.65
Soy protein concentrate ^4^	11.49
Soy protein isolate	91.05
Shoyu (soy sauce)	1.18
Soy beans, roasted	148.5
Soy beans, raw	154.53
Yuba/foo jook	44.67
Soy milk ^5^	0.7–10.73
Sufu	13.75
Tempeh	3.82
Tofu ^6^	13.1–34.78
Okara	9.39
Fuyu	45.51
Soybean oil	0

^1^ From USDA Database for the Isoflavone Content of Selected Foods [64]. ^2^ American, Cheddar, Monterey Jack, Mozzarella, Parmesan, Swiss, unspecified. ^3^ by aqueous washed. ^4^ by alcohol extraction. ^5^ Original, from soy isolate, unflavours, flavours, non-fat, low-fat, full fat, fortified, unfortified. ^6^ regular, extra firm, firm, silken, soft, yogurt, raw, pressed, steamed, cooked, dried, braised, fried, smoked.

**Table 7:** Soy foods description ^1^.

Item	Description
Tofu	Soaking and heating procedures with addition of protein coagulants such as calcium sulphate to soymilk. Pressed soy curd can undergo smoking or marinating processes.
Soy milk	Water extraction of hulled and crushed soy beans. Boiling after filtration of raw milk removes beany flavour.
Tempeh	Dehulled soy beans fermented with *Rhizopius oligosporus*.
Natto	Soy beans fermented with *Bacillus natto* and *Bacillus subtilis*.
Sufu	Tofu fermented with *Actinomucor elegans*.
Edamame	Immature, green soy beans.
Miso	Fermented soy beans with *Aspergillus orzae* or *Aspergillus soyae*.
Yuba	Drying of skin (film) formed in soymilk production during boiling.
Soy Sauce	Soy beans or soy flakes fermented with different bacteria, yeast or treated with isolated enzymes. Pressing and filtration after fermentation is needed to extract aqueous phase.
Textured vegetable protein	Extrusion and cooking of soy flour, full fat or defatted, in moisture and temperature controlled conditions.

^1^ Adapted form references [13,18].

## Abbreviations

ADA	American Dietetic Association
APP	alternate protein product
BV	biological value
CHD	coronary heart disease
CVD	cardiovascular disease
DIAAS	digestible indispensable amino acid score
EFSA	European Food Safety Authority
EPIC	European Prospective Investigation into Cancer and Nutrition
ER	oestrogen receptor
FAO	Food and Agriculture Organization
FDA	Food and Drug Administration
GMO	genetically modified organism
HDL	high density lipoprotein
HHL	health-conscious lifestyle
LC-MS/MS	liquid chromatography tandem-mass spectrometry
LDL	low density lipoprotein
MRI	magnetic resonance imaging
NAHANES	National Health and Nutrition Examination Survey
NPU	net protein utilization
OR	odds ratio
PDCAAS	protein digestibility corrected for amino acid score
PER	protein efficiency ratio
PSA	prostate specific antigen
PUFA	polyunsaturated fatty acids
SERM	selective oestrogen receptor modulator
SPI	soy protein isolate
STEAR	selective tissue estrogenic activity
TSH	thyroid-stimulating hormone
TVP	textured vegetable protein
USDA	United States Department of Agriculture
WHO	World Health Organization
*w*/*w*	weight per weight
